# Sequential degradation of raw vinasse by a laccase enzyme producing fungus *Pleurotus sajor-caju* and its ATPS purification

**DOI:** 10.1016/j.btre.2019.e00411

**Published:** 2019-12-13

**Authors:** Joberson Alves Junior, Yago Araujo Vieira, Ianny Andrade Cruz, Débora da Silva Vilar, Mario M. Aguiar, Nádia Hortense Torres, Ram Naresh Bharagava, Álvaro Silva Lima, Ranyere Lucena de Souza, Luiz Fernando Romanholo Ferreira

**Affiliations:** a Graduate Program in Process Engineering, Tiradentes University, Av. Murilo Dantas 300, Farolândia, 49032-490, Aracaju, SE, Brazil; b Division of Molecular Biology - Biocenter, Innsbruck Medical University, A-6020, Innsbruck, Austria; c Institute of Technology and Research, Av. Murilo Dantas 300 - Prédio do ITP, Farolândia, 49032-490, Aracaju, SE, Brazil; d Laboratory for Bioremediation and Metagenomics Research (LBMR), Department of Microbiology (DM), Babasaheb Bhimrao Ambedkar University (A Central University), Vidya Vihar, Raebareli Road, Lucknow, 226 025, Uttar Pradesh, India

**Keywords:** Biomass, biological treatment, decolorization, inducers, laccase purification

## Abstract

This study evaluated simultaneously the raw vinasse degradation, an effluent from the sugar-alcohol industry, the laccase production by *Pleurotus sajor-caju* and its purification using aqueous two-phase systems (ATPS). To improve laccase production, different concentrations of inducers (ethanol and CuSO_4_) were added. The higher laccase production promoted an increase of 4-fold using 0.4 mM of CuSO_4_ as inducer, with maximum enzymatic activity of 539.3 U/L on the 3^rd^ day of fermentation. The final treated vinasse had a decolorization of 92% and turbidity removal of 99% using CuSO_4_. Moreover, the produced laccase was then purified by ATPS in a single purification step, reaching 2.9-fold and recovered ≈ 99,9 %, in the top phase (PEG-rich phase) using 12 wt% of PEG 1500 + 20 wt% of citrate buffer + enzyme broth + water, at 25 °C. Thus, an integrated process of vinasse degradation, laccase production and purification with potential industrial application was proposed.

## Highlights

• Vinasse degradation and laccase production by *Pleurotus sajor-caju* were performed;
• Laccase activity induction by copper sulfate and ethanol in raw vinasse as substrate was confirmed;
• Fermentation time to maximum laccase activity was reduced to just 3 days when cooper sulfate was used as inducer;
• The use of laccase inducers does not interfere with decolorization and turbidity removal;
• Aqueous two-phase systems reached 2.88-fold in laccase purification, with recovery of ∼ 99.9% to upper phase (PEG-rich phase).

## Graphical abstract

**Figure:**
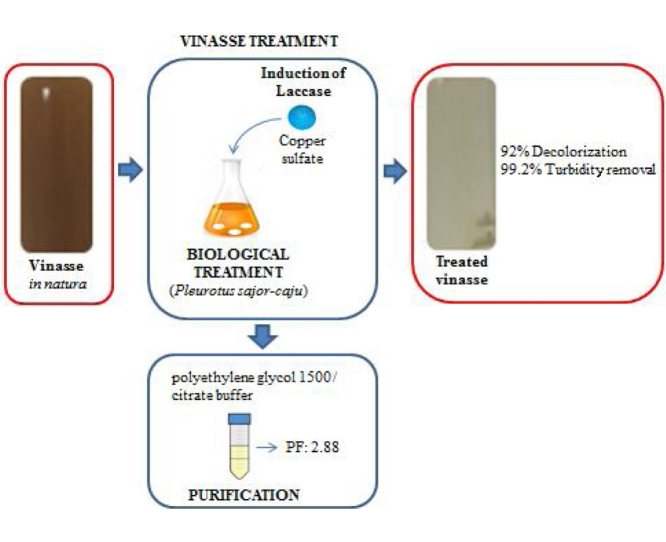

## 1 Introduction

The sugarcane industry is an important economic segment and well developed in Brazil, making sugarcane ethanol the most successful alternative fuel program ever developed in the world [1]. According to the Brazilian National Supply Company [2], in 2017, approximately 28 billion liters of ethanol were produced in the country and with a continuously increase in the upcoming years. For each liter of ethanol obtained, between 9 and 14 L of the residue vinasse is produced [3]. Raw vinasse is mainly composed of water, inorganic minerals, suspended solids and organic pollutants such as phenolic and melanoidin compounds. It has a brown color, a corrosive low pH (3.5 – 5.0), a high chemical oxygen demand (70 – 150 g/L) and biochemical oxygen demand of 35 – 50 g/L. These characteristics make vinasse a complex residue to decompose [4] and its intensified use in agriculture cause nutrient saturation and pH increase, but it still is used as a fertilizer for sugarcane crop production.

When discarded in water bodies, vinasse causes the dissolved oxygen to be consumed faster, compromising the aquatic biota [5]. Vinasse can also lixiviate into ground water, reaching and contaminating underground aquifers [6]. Therefore, it is important to the discarded vinasse undergo treatment before its release into the environment and/or to use it to obtain other products of economic interest. Vinasse degradation by microorganisms has proved to be an efficient form of treatment [7], specially by using the basidiomycete *Pleurotus sajor-caju* [8]. This biodegradation alternative may be further exploited since the fungus produces and excretes enzymes of commercial interest such as laccase (EC 1.10.3.2).

Lignin-modifying enzymes (LMEs) are types of enzymes produced by fungi that catalyze the breakdown of different organic and inorganic substrates. These enzymes have a great potential in biotechnological applications, such as in the food industry, delignification of cellulosic compounds, paper bleaching, degradation of synthetic dyes and pesticides in the soil [9], and in the breakdown against several micropollutants including already recognized endocrine disrupting chemicals at their natural residual concentrations [10].

Furthermore, the laccase production has attracted attention with the use of inducers as a strategy to increase its production using *Ganoderma lucidum* [11] and *Pleurotus ostreatus* fungi [12]. Aromatic compounds, such as pyrogallol and ferulic acid have been effective in stimulating laccase production by *P. sajor-caju* [13]. However, most of these compounds are harmful to humans and have a high cost, making it problematic in industrial applications. Some authors report that different alcohols can be more adequate and have an economic advantage to inducing production [11,14]. Another well-known inducer is copper, as it increases the laccase production by fungi due to the affinity of copper atoms at its catalytic site [15,16].

Depending on the application and commercial production, laccase must through a purification process. Among the most common techniques applied for laccase purification are filtration followed by membrane ultrafiltration [13], precipitation followed by dialysis [17], freezing and thawing followed by centrifugation [18] and by chromatographic techniques [19]. These techniques require several steps, which can cause increase in the final cost and loss of enzyme activity [20].

Liquid-liquid extraction by aqueous two-phase systems (ATPS) can then be used as an alternative for the separation and purification of biomolecules such as proteins, enzymes and nucleic acids. This methodology has a low cost and guarantees excellent quality levels of purity and enzymatic activity [21,22]. ATPS is formed when two water-soluble compounds are mixed above its critical concentrations, resulting in two immiscible phases [23]. The separation process is the result of specific interactions between the solute and the phase-forming components. ATPS based on polymers and salts have been well explored for the purification of different enzymes [19,20,47], and a series of new constituents based on ionic liquids are being investigated [24]. However, the chemical cost is always important for any industrial separation process, and polymer ATPS are still recommended for commercial applications [19].

Therefore, it is important to consider the large volume of available biomass from the sugar-alcohol industry, that may contribute in adding commercial value to new sugarcane production chains as well as strengthening the creation of a chemical industry in a green economy [25]. In this sense, this work evaluated the laccase production from *P. sajor-caju* during the sugarcane vinasse biodegradation. For this purpose, different concentrations of inducers such as ethanol and copper sulfate were investigated. Moreover, it was proposed the use of ATPS based on polymers PEG (polyethylene glycol) and the study of various conditions, namely the polymer molecular weight and the constituent’s concentration in terms of their effects on the laccase purification.

## 2 Materials and methods

This section describes the materials and main procedures used in the production, induction and purification of *P. sajor-caju* laccase.

### 2.1 Vinasse

The sugarcane vinasse used in this work was collected at the São José do Pinheiro power plant (10º46'10.8 "S, 37º12'48.3" W), located in the city of Laranjeiras, State of Sergipe, Brazil. The residue was collected in 5 L containers and kept refrigerated at −4 °C.

### 2.2 Microorganism and Fermentation

*P. sajor-caju* was obtained from the São Paulo Botanical Institute, Brazil (under the code CCIBt 020) and kept refrigerated at 4 °C in Petri dishes containing MEA (malt extract agar) medium. The fungus was inoculated in a 250 mL Erlenmeyer flask containing 100 mL of vinasse with pH 6.0, and previously autoclaved at 121 °C and 1 atm for 15 minutes. Upon cooling, these flasks were inoculated with three discs (1 cm in diameter) of culture medium containing the fungus mycelia. The flasks were incubated for 15 days in shaker at 180 rpm and 28 °C (±2 °C), under no light conditions, and samples were collected every 3 days.

For the enzyme induction test, different concentrations of inducers were added to the vinasse containing medium and fermentation was carried out for 15 days. The inducers tested were ethanol (at 1, 2 and 3%, v/v) and CuSO_4_ (at 0.4, 0.6 and 1.0 mM).

### 2.3 Vinasse decolorization and Turbidity removal

The supernatant was filtered and absorbance (abs) measured (λ =475 nm) using a Varian Cary 50 spectrophotometer. To calculate vinasse decolorization the following equation was applied:

(1) Decolorization (%) = [(abs_initial_ - abs_final_) / abs_initial_] x 100

Turbidity was measured using a portable HI 98703 turbidimeter and values expressed in NTU (Turbidity Nephelometric Units).

### 2.4 Mycelial biomass quantification and Protein quantification

The biomass was determined by the gravimetric method by drying it at 45 °C until constant weight was obtained.

The total protein concentration was determined by the Bradford method (1976) and a calibration curve was determined using bovine serum albumin as standard and values expressed as mg/L.

### 2.5 Enzymatic activity

Laccase activity was determined at 30 °C using a mixture of 0.3 mL of 0.05 M citrate-phosphate buffer (pH 5.0); 0.1 mL of syringaldazine solution, which is a reaction indicator, and 0.6 mL of the crude enzyme solution. Syringaldazine oxidation was measured by monitoring the increase in absorbance at a wavelength of 525 nm after 10 minutes of reaction. A unit of enzyme activity was defined as the amount of enzyme required to oxidize 1 μmol of syringaldazine per minute, with laccase activity expressed as unit of enzyme per liter of enzyme extract (U/L) [26].

### 2.6 Purification

The ATPSs were prepared in graduated centrifuge tubes by weighing the appropriate amounts of PEG, citrate buffer, distilled water and fermented broth (item 2.2) containing laccase. The tubes were shaken and centrifuged at 3000 rpm for 20 minutes and placed in a thermostatic bath at 25 °C (±1 °C) and atmospheric pressure for 12 hours to achieve a thermodynamic equilibrium. The system top and bottom phases were carefully collected with a precision needle. All experiments were performed in triplicate and purification parameters were determined.

The partition coefficient was defined as the protein concentration or enzymatic activity in the top phase, divided by the corresponding value in the bottom phase. K_P_ is the protein partition coefficient and K_E_ is the enzyme partition coefficient, which are found following the equations:

(2) *K_P_* = C_T_ / C_B_

(3) *K_E_* = EA_T_ / EA_B_

where C_T_ is the total protein concentration (mg/L) in the top phase, C_B_ is the total protein concentration in the bottom phase, EA_T_ is the enzymatic activity (U/L) in the top phase and EA_B_ is the enzymatic activity in the bottom phase.

To evaluate the purification process, the specific enzyme activity (SA, U/mg protein) was calculated by equation (4), the enzyme recovered in the top (R_ET_,%) and bottom (R_EB_, %) phases were calculated by equations (5) and (6), respectively, after calculating the volumetric ratio between phases (R_V_), and finally the purification factor (PF), calculated according to equation (7).

(4) *SA* = EA / C

(5) *R_ET_* = 100 / (1 + (1 / (R_V_K_E_)))

(6) *R_EB_* = 100 / (1 + K_V_K_E_)

(7) *PF* = SA / SA_i_

Where the purification factor (PF) was calculated using the ratio between the specific activity (SA) in the top or bottom phases (depending on the stage in which the enzyme is concentrated) and the laccase specific activity (SA*i*) after the fermentation process.

## 3 Results and Discussion

### 3.1 Laccase production and induction

Cultivation of *P. sajor-caju* in vinasse was carried out for 15 days at 28 °C and 180 rpm of agitation. Due to the adaptation conditions of the *P. sajor-caju* fungus in the medium, the pH was adjusted to 6.0 since an acidic acid pH could cause enzyme denaturation [27]. In previous studies, the adjustment in the vinasse pH was shown to increase the residue degradation [7,8].

**Fig. 1:**
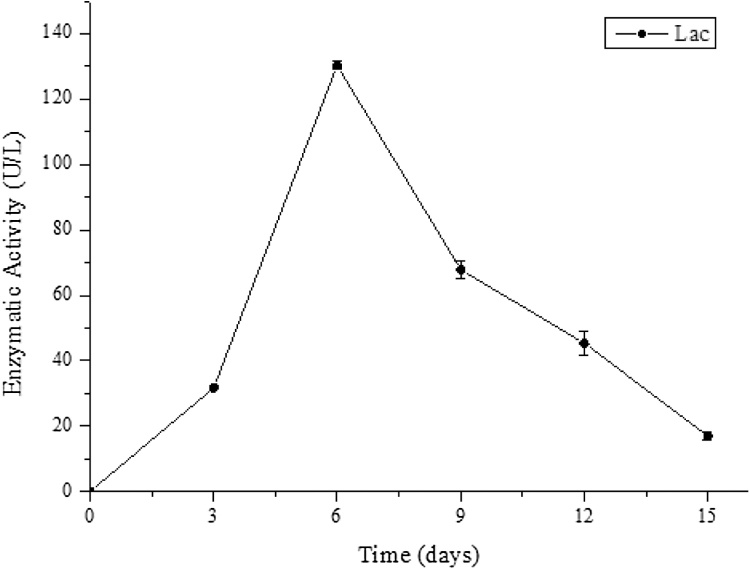
Laccase activity (U/L) (●) of P. sajor-caju during 15 days of cultivation at 28 ºC (± 2 ºC) and 180 rpm in raw vinasse.

The laccase production is shown in Fig. 1, where we observe that the highest production occurred on the 6^th^ day of cultivation with an activity of 130.3 U/L. For the same fungus and medium (100% vinasse) and through semi-solid fermentation Aguiar et al. [28] obtained a maximum laccase activity of 10.53 U/L during the first 6 days. Ferreira et al. [7] using submerged fermentation, obtained production of 400 U/L on the 9^th^ day of cultivation and Vilar et al. [8] 400 U/L on the 12^th^ day. These differences between enzymatic activities are due to factors such as the chemical composition of different vinasse batches, the processes for the residue generation and whether molasses was present in the broth.

Laccase production by *P. sajor-caju* may vary depending on the growth conditions. Fokina et al. [16] yielded 70 U/L production in 7 days of culture in YGA (yeast extract-agar-glucose) medium at 28 °C and 120 rpm shaking. The effect of temperature and pH on the production of *P. sajor-caju* laccase was studied in modified Kirk medium, with varying temperature between 20 and 35 °C and pH between 4 and 6, and the optimum conditions were 30 °C, pH 6 and 135 rpm, with enzymatic activity of 620 U/L [29]. Another study used a liquid growth medium composed of minerals, where laccase activity of approximately 17 U/L was obtained on the 8^th^ day of culture at 30 °C [30]. On an industrial scale, Liu et al. [31] produced laccase from *Pycnoporus* sp. reaching 80 U/mL in a 5-ton bioreactor using a medium composed of maltose, yeast extract and peptone. Consequently, laccase production may vary according to culture conditions, for example the pH, incubation temperature, fermentation time, agitation, culture medium, presence of inducers and the species of microorganism used [32,33].

#### 3.1.1 Laccase induction by copper sulfate

**Fig. 2:**
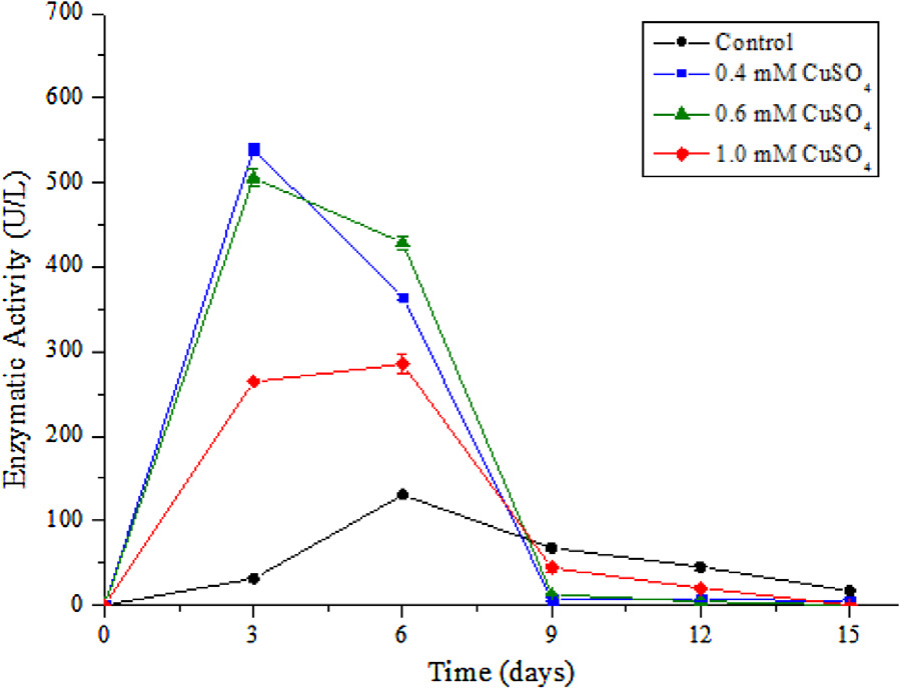
Laccase activity (U/L) of P. sajor-caju during 15 days of cultivation at 28 ºC (± 2 ºC), 180 rpm and induced by CuSO4 in raw vinasse. Control (●), 1.0 mM (), 0.6 mM ( and 0.4 mM ().

Inducers are critical to increase enzyme production [12] and copper is well-known in the literature for elevating laccase activity considerably [16]. Concentrations of 1.0 mM and 0.6 mM CuSO_4_ (Fig. 2) resulted in maximal enzymatic activities of 285 and 505.5 U/L, respectively, on the 6^th^ and 3^rd^ day of culture. The highest laccase activity occurred on day 3 with 539.3 U/L, in the concentration of 0.4 mM CuSO_4_, a 4-fold increase difference from the control sample, which consisted of 100% vinasse medium. This supports the relevance in the use of laccase inducers to reduce fermentation time and the production costs. Nonetheless, this result corroborates with the data found by Manavalan et al. [11] for laccase production by *Ganoderma lucidum*, where a 7.5-fold increase in enzyme activity was achieved in nutrient medium with starch, supplemented with 0.4 mM CuSO_4_.

Although the precise mechanisms are still under investigation, copper promotes production through the transcription of fungal laccase genes [34]. Another hypothesis is related to the copper atoms present in the catalytic site of the enzyme. By adding more copper to the medium, it acts as an enzymatic cofactor, increasing the number of functional groups associated with enzymatic catalysis and consequently increasing laccase activity [34]. For the fungus studied, lower copper concentrations gave a higher activity. Although copper is essential for inducing laccase production in basidiomycetes, there is an ideal amount needed for each species and an excessive amount leads to reduction in enzymatic activity. One of the explanations for this is that at high concentrations, copper acts as a potent inhibitor of fungus growth [9,35]. This was confirmed by Patrick et al. [29], where concentrations between 0.1 and 2.0 mM for *P. sajor-caju* laccase production were tested and the best results were obtained at concentrations of 0.1 and 0.2 mM. Another author obtained similar results, reaching high yields of *Lentinus polychrous* laccase in concentrations of 0.1 and 0.3 mM CuSO_4_ and low yields at concentrations of 1.0 and 3.0 mM [15].

Similar results in laccase production in *P. sajor-caju* were found using 0.3 mM CuSO_4_ as inducer, where enzymatic activity was increased by 3.7 fold [29]. In another study, laccase production increased from 70 to 185 U/L by adding 1.0 mM CuSO_4_ at the 3^rd^ day of inoculation [16]. The present work demonstrates an increase of 4-fold in enzymatic activity by using 0.4 mM CuSO_4_. The maximum peak of activity was observed with a reduced fermentation time from 6 to 3 days, in relation to the control sample (100% vinasse).

#### 3.1.2 Laccase induction by Ethanol

The use of alcohols, such as ethanol, has been reported as a substitute for aromatic compounds (pyrogallol and ferulic acid) in inducing laccase production by *P. sajor-caju*, and making it more cost efficient [11].

**Fig. 3:**
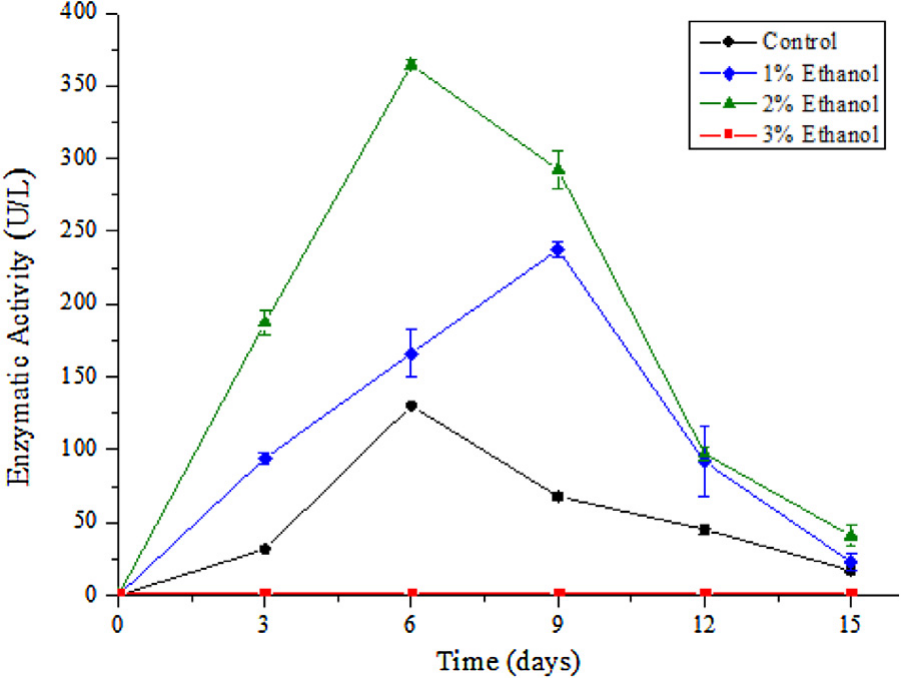
Laccase activity (U/L) of P. sajor-caju during 15 days of cultivation at 28 ºC (± 2 ºC), 180 rpm and induced by ethanol in raw vinasse. Control (●), 3% (v/v) , 2% (v/v)  and 1% (v/v) .

A highest laccase activity (237.5 U/L) was observed on the 9^th^ day of incubation with 1% ethanol addition. With a 2% ethanol addition, laccase activity increased to 364.5 U/L (2.8-fold) on the 6^th^ day of cultivation. When ethanol concentration is increased to 3%, no laccase activity is detected. While the concentration of 1 and 2% of ethanol improved the enzyme activity and the biomass production, 3% of ethanol presented high toxicity on the microorganism growth and consequently no enzyme activity (Fig. 3).

In Manavalan et al. [11], concentrations up to 5% ethanol were used to induce laccase production from the fungus *G. lucidum*. However, it is worth noting that different vinasse samples have different residual ethanol amounts as a result from the distillation process. This in part can influence the final ethanol concentration explaining why 3% ethanol inhibited fungal growth and laccase production in this study.

Although ethanol is proposed as a laccase inducer, little is known about its mode of induction. Some authors suggest that ethanol can induce enzyme genes through oxidative stress, and/or cause membrane rupture, promoting enzyme secretion [36,37]. One factor that may affect the ethanol induction is the nitrogen found in the culture medium. Hernández et al. [14] induced the laccase production from *Pycnoporus sanguineus* using 3 g/L of ethanol and a source of simple (mineral) or complex (organic) nitrogen. Laccase activity of 31 U/L was obtained for ethanol and organic nitrogen source and 1.6 U/L for ethanol and mineral nitrogen source, showing that laccase production depends on the nitrogen source as well. Since vinasse is a complex organic form of nitrogen, the results here obtained are supported from the ones obtained by Hernández et al. [14].

For *G. lucidum* laccase production was induced by ethanol in all tested concentrations (1, 2, 3, 4 and 5% v/v). The highest production (2.5 U/mL, a 14-fold increase) was achieved with 3% ethanol in basal medium for 15 days at 30 °C, also demonstrating a high ethanol tolerance [11].

### 3.2 Mycelial biomass

**Fig. 4:**
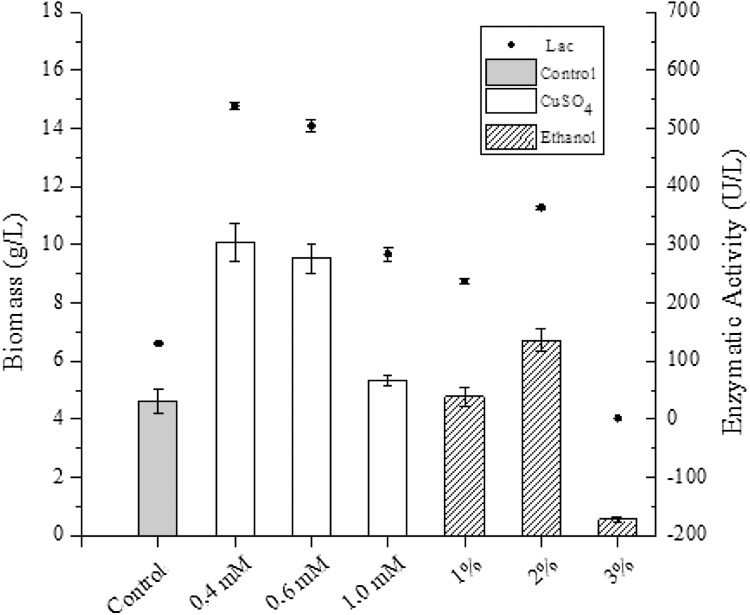
Biomass and laccase activity (U/L) of *P. sajor-caju* during 15 days of cultivation at 28 °C (± 2 °C) and 180 rpm in raw vinasse medium and inducers.

Fungal biomass is a versatile source for food additives, as well as for extracting antimicrobial compounds, aromas, polysaccharides and antioxidants (Kirsch et al., 2016). Within this scope, the cultivation of *P. sajor-caju* in vinasse medium with CuSO_4_ as an inducer resulted in a biomass accumulation of 10.08 g/L. Fig. 4 represents biomass concentration in different medium conditions. It is observed that the laccase activity is proportional to the biomass accumulation [8,31].

In a study that evaluated biomass production by *P. sajor-caju* using a medium containing vinasse and sugarcane bagasse, during the 30 days of fermentation, found 117.87 g/L of fungal biomass produced. This value was attributed to the highly nutritive nature of the medium with bagasse [28]. Using the same fungus species, Vilar et al. [8] obtained a biomass production of 22.06 g/L in medium containing vinasse enriched with glucose, showing the importance of glucose for biomass production. In another study, the relationship between biomass production and laccase was studied by cultivating the fungus in mineral solution where 5.2 g/L of biomass was associated with a high laccase activity (40 U/mL) [38].

### 3.3 Vinasse decolorization and turbidity removal

**Fig. 5:**
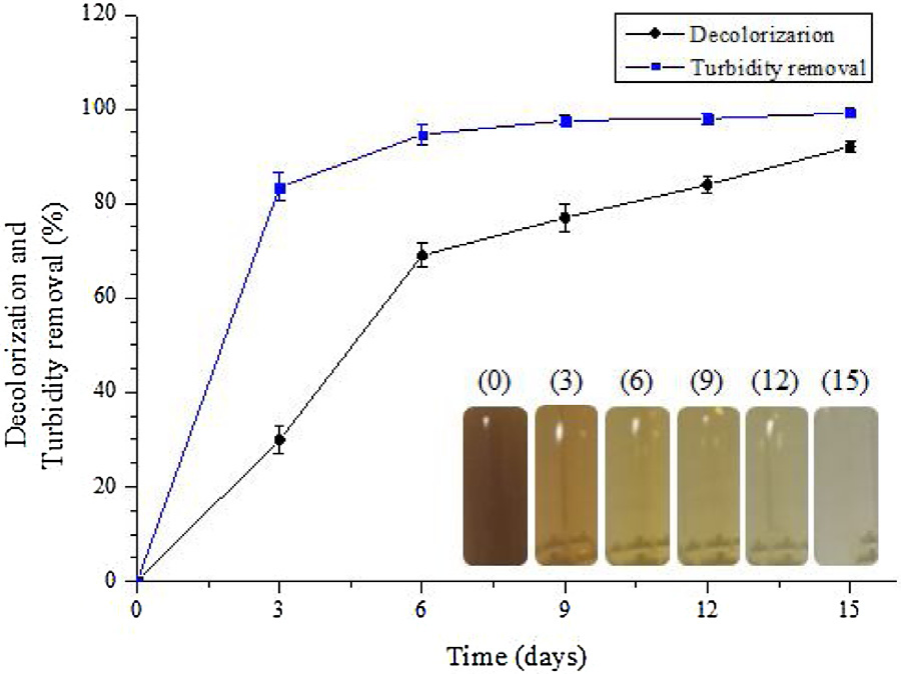
Percentage decolorization and turbidity removal of raw vinasse following treatment with *P. sajor-caju* for 3, 6, 9, 12 and 15 days, at 28 °C (± 2 °C) and 180 rpm.

Vinasse is highly polluting and contains tannic acid, humic acid and melanoidines, which give its characteristic dark color. These phenolic compounds are recalcitrant biopolymers derived from the Maillard reaction where amino acids react with reducing carbohydrates [4,39]. Therefore, it is fundamental to apply an adequate treatment to reduce its color and turbidity for a safe discard in the environment or re-utilization in industrial processes. In this study, the processes of decolorization and turbidity removal begins upon the 3^rd^ day of fermentation (Fig. 5). The highest values achieved were 92% and 99.2% for color removal and turbidity, respectively, after the 15^th^ day of culture in medium without addition of inducers.

The values of decolorization and removal of turbidity presented in this study are close to those already described in the literature [7]. The decolorization and turbidity reduction of vinasse is due to the activity of enzymes, which attack the recalcitrant compounds responsible for this characteristic color [8]. These results show the consistency of our data using *P. sajor-caju* for the vinasse biological treatment.

Vinasse decolorization was better achieved in the media containing inducers (from 81 to 92%). The same occurred for turbidity removal, whereas all CuSO_4_-induced samples obtained were between 97.8 % and 99%. Those induced by 1% and 2% ethanol obtained 92.6 and 97.8% turbidity reduction, respectively. The 3% ethanol concentration did not display any decolorization or turbidity removal of the medium in *P. sajor-caju* presence. The probable reason, as stated before, might be due that alcohol becomes toxic to *P. sajor-caju* at this concentration, thus halting the fungus to develop.

Studies report on the decolorization and turbidity removal from vinasse using other treatments and microorganisms. Values of 89.7% decolorization and 79.2% turbidity removal were obtained by treating the residue with the microorganisms present in activated sludge and in several process steps (fermentation with a high flow system, filtration, chemical flocculation, fermentation with a low flow system, neutralization and disinfection) [40]. Thanapimmetha et al. [41] obtained 88.5% color removal from vinasse by using an electro-Fenton process. Rodríguez et al. [42] used an anaerobic fluidized bed reactor to treat vinasse through microbial communities (bacteria and archaea) while the fungus *Phanerochaete chrysosporium* was used by Dahiya et al. [43] to obtain 80% decolorization. This color removal is attributed to melanoidin degradation, and for biological treatments, this is due to the enzymatic activity of the microorganisms employed.

The results show that the addition of CuSO_4_ and ethanol as laccase inducers does not interfere in the melanoidin degradation. Synthesis and excretion of laccase by the fungus continues to occur, consequently breaking down these complex compounds in the vinasse. The *P. sajor-caju* efficiency in decoloring vinasse and its capacity to degrade recalcitrant compounds was proven in this work, corroborating with others in the literature [7,8], where high rates of decolorization and reduction of turbidity were achieved.

### 3.4 Purification using ATPS

Systems based on PEG and citrate buffer were used to purify laccase from vinasse degradation by *P. sajor-caju*. The systems were initially prepared using 10 wt% of PEG 4000 + 15 wt% of citrate buffer + 30 wt% enzyme broth + water at 25 °C. The data of composition for the biphasic systems formed by PEG and citrate buffer were previously described in the literature [44].

The laccase partition at different pH values (5.0, 6.0 and 7.0) was investigated in ATPS (salt-rich phase). Enzymes are formed by amino acid residues in the active site that have acidic or basic properties and are important for catalysis. Changes in pH values can affect these residues and cause enzymatic denaturation. Table 1 shows the effect of pH on the enzymatic activity of recovered laccase in the top and bottom phases of the ATPS. The most active laccase was observed at pH 6, with 507.2 ± 24.3 U/L for the PEG-rich phase. Higher and lower pH values led to a significant decrease in laccase activity and purification factor. This same behavior was observed by Silvério et al. [45] regarding the stability of commercial laccase from *T. versicolor* with different pH buffers.

**Table 1:** Laccase activity (U/L) at the top and bottom phases at different values of pH (5.0, 6.0 and 7.0) of the ATPS composed of 10 wt% of PEG 1500 + 15 wt% of citrate buffer + water, at 25 ± (1.0) ºC.

pH	Laccase activity (U/L)	
	Top phase	Bottom phase
5.0	27.4 ± 5.8	21.9 ± 4.6
6.0	507.2 ± 24.3	195.4 ± 18.0
7.0	438.7 ± 20. 6	78.8 ± 10.3

In addition, it is possible to observe the laccase partition to the PEG-rich phase (*K_E_* > 1). The isoelectric point (*pI*) of the enzyme laccase is 4.5 [46], this suggests that the bottom-phase (salt-rich phase) at pH 6, amino acid residues of the enzyme are partially negatively charged, which may justify the laccase partition into top phase (PEG-rich). This phenomenon was observed by other authors [19,43]. The influence of pH (5.8 to 8.0) on the partition of phenylalanine dehydrogenase in PEG/ammonium sulfate ATPS was investigated by Mohamadi et al. [47]. The pH increase above the *pI* of the protein, the net negative charge of the protein increased, favoring the interaction between the protein and the PEG-rich phase, consequently increasing the partition coefficient. The further steps of purification and optimization were then conducted in ATPS using pH 6.0.

**Fig. 6:**
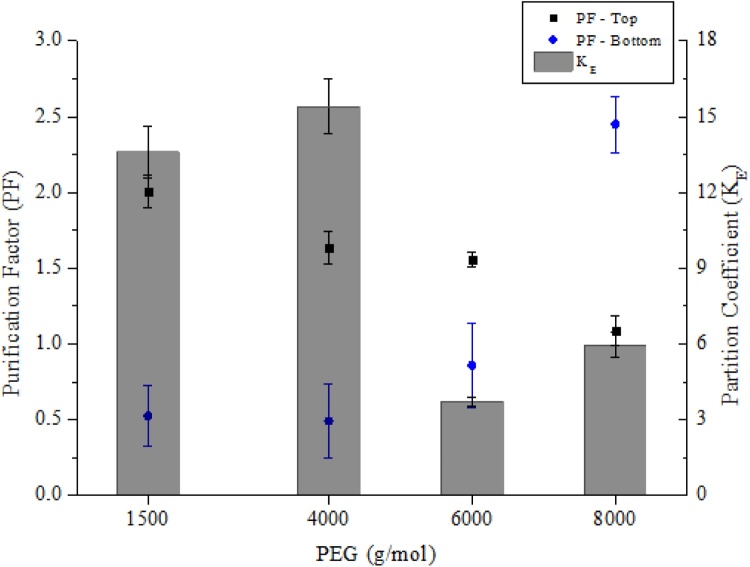
Partition coefficient (K_E_) and purification factor (PF) of laccase at the top/bottom phases of the PEG system at different molecular weights (1500, 4000, 6000 e 8000 g/mol) and citrate (pH 6.0) at 25 °C.

To improve the purification of laccase from *P. sajor-caju*, different molar mass of PEG were investigated (1500, 4000, 6000 and 8000 g/mol). The systems were prepared at fixed concentrations of 10 wt% of PEG + 15 wt% of citrate buffer (pH 6.0) at 25 °C. The enzyme partition coefficient (*K_E_*) and purification factor (*PF*) of laccase are showed in Fig. 6.

The laccase partition to the top phase (PEG-rich phase) of the system was observed for all investigated systems. PEG of smaller molecular weight (1500 and 4000 g/mol) resulted in greater migration of laccase to the top phase compared to PEG of larger molecular weight, such as 6000 and 8000 g/mol (Fig. 6). These results led to the increase of the laccase purification factor (*PF*) in the top phase for the smallest weight polymers, especially for the PEG 1500 system with a PF ≈ 2.1, while for the system with PEG 8000 a *PF* ≈ 1.0 was found. According to Ratanapongleka [48], increasing the PEG molar mass results in an exclusion effect, causing a reduction in the free volume and making the polymer to a more hydrophobic and compact conformation. This makes it difficult to partition the protein to the top phase. The results corroborate with those described by Sánchez-Trasviña et al. [49], where *Trametes versicolor* laccase had a preference for the top phase of the system formed by PEG and potassium phosphate, and by increasing the PEG molar mass, the recovery of laccase in the PEG-rich phase was decreased. Furthermore, polymers of high molecular weight strongly increase the viscosity of the medium that results in difficulties to enlarge the scale and in effect of denaturation to proteins, therefore, not suitable for ATPS.

In addition to molecular weight, the choice of the PEG concentration has the significant effect on the enzyme partition in ATPS. The effect of polymer concentration was investigated for system composed with PEG 1500 (10 – 18 wt%) + citrate buffer at pH 6.0 (15 wt%). Table 2 shows the laccase purification and recovery factors at the top and bottom phases. The hydrophobic interaction between the PEG and the enzyme surface increases with increasing polymer concentration up to the 12 wt% of PEG and PF ≈ 2.3. PEG concentrations above 12 wt% made the purification factor decrease, probably because of the increase in viscosity and interfacial tension between the phases, making it difficult the migration of laccase into the top phase [50]. Kirincic and Klofutar [51] showed that the increase in molecular weight of PEG (400 - 20000) increased the viscosity of the aqueous PEG solution from 4.41 cm^3^/g to 42.02 cm^3^/g, respectively.

**Table 2:** Purification factor (PF) and recovery of laccase in the top (*R_ET_*) and bottom (*R_EB_)* phase from *Pleurotus sajor-caju*, for systems based in wt% PEG 1500 + wt% citrate buffer (pH 6.0) + water, at 25 ± 1.0 °C.

PEG 1500 (wt%)	Salt (wt%)	*R_ET_* (%)	*R_EB_* (%)	PF top	PF bottom
10	15	86.53 ± 3.33	13.47 ± 3.33	2.01 ± 0.11	0.04 ± 0.01
12		99.81 ± 0.01	0.19 ± 0.01	2.31 ± 0.13	0.02 ± 0.01
15		99.46 ± 0.19	0.54 ± 0.19	2.18 ± 0.08	0.04 ± 0.01
18		99.18 ± 0.04	0.81 ± 0.04	1.95 ± 0.10	0.05 ± 0.02
12	15	99.81 ± 0.08	0.19 ± 0.08	2.31 ± 0.13	0.02 ± 0.01
	18	99.92 ± 0.02	0.12 ± 0.02	2.65 ± 0.11	0.04 ± 0.01
	20	99.92 ± 0.01	0.08 ± 0.01	2.88 ± 0.08	0.03 ± 0.01
	22	99.58 ± 0.02	0.42 ± 0.02	0.94 ± 0.02	0.01 ± 0.01
	24	99.48 ± 0.04	0.53 ± 0.04	0.71 ± 0.13	0.01 ± 0.01

The effect of citrate buffer (pH 6.0) concentration (15 – 24 wt%) was also investigated (Table 2). The increase in salt concentration promoted both recovery and purification of the laccase to the top phase of the system. The enzyme recovery changed of 99.81 % to 99.92 % and a PF of 2.31 to 2.88, from the systems with 15 wt% to 20 wt% of citrate buffer (pH 6,0) + 12 wt% of PEG 1500 + water, at 25 °C. The effect of salt concentration on the system was clearly influenced by the salt-out ability of the salt used [52], whereby the enzymes are partially hydrated with increasing salt concentration and easily expelled to the opposite phase (PEG rich phase). At concentrations above 20 wt% PF decreased considerably, probably due to the increase in density at the salt-rich phase [50]. Mayolo-Deloisa et al. [50] obtained a purification factor of 2.48 and recovery of 95% when using a similar system consisting of PEG 1000 and potassium phosphate to recover laccase produced by *Agaricus bisporus.* Ratanapongleka (2012) purified laccase from *Lentinus polychrous* to 1.98-fold, with a 99% recovery, in PEG 4000 and potassium phosphate systems. *P. sapidus* laccase was also purified to 1.68-fold, a recovery yield of 105% for the top phase of a PEG 3000 and phosphate system [21]. More recently, Rajagopalu et al. [22] obtained a purification factor of 8.03 fold and an recovery of 99.4% of laccase from *Hericium erinaceus* in the bottom phases of ATPS formed by PEG 8000 + phosphate buffer.

This developed process for the purification of laccase produced from vinasse substrates using PEG is effective and suppresses enzyme denaturation. In addition, the ATPS makes use of citrate salt making it a more sustainable process due to the biodegradable and non-toxic nature of citrate.

## 4 Conclusion

*Pleurotus sajor-caju* was used to produce laccase enzyme with simultaneous degradation of vinasse to ethanol production. The presence of inducers, during the degradation and consequently laccase production, was significantly influenced by the presence of the copper ion. The maximum activity of the laccase enzyme was ≈ 539.3 U / L on the 3^rd^ day of fermentation with 0.4 mM CuSO_4_. Under these conditions, the final treated vinasse had a ≈ 92% discoloration and ≈ 99% turbidity removal. In addition, the produced laccase was then purified by ATPS in a single purification step. Factors such as composition and pH of the phases (12 wt% PEG 1500 + 20 wt% citrate buffer (pH 6.0) + enzyme broth + water) were crucial for the separation process. The laccase was then purified ≈ 2.9-fold, with recovery of ≈ 99.9% to the upper phase (PEG-rich phase).

Therefore, this study successfully used inducers to stimulate enzymatic production and reduce the vinasse polluting potential, thus adding value to the by-product. In addition, these results also indicate that the use of ATPS is a promising method for the purification of laccase from *P sajor-caju* and this partially purified extract can be a suitable biocatalyst for a large number of industrial processes.

## Declaration of Competing Interest

The authors declare that there is no conflict of interest. Further, the work reported in this manuscript is original and has not been published previously. All the authors have seen and approved the final version submitted to journal and the consent from all the authors is given for its publication in “Biotechnology Reports”.
